# Racial Disparities in the Use of Cardiac Revascularization: Does Local Hospital Capacity Matter?

**DOI:** 10.1371/journal.pone.0069855

**Published:** 2013-07-16

**Authors:** Suhui Li, Arnold Chen, Katherine Mead

**Affiliations:** 1 Department of Health Policy, George Washington University, Washington, District of Columbia, United States of America; 2 Mathematica Policy Research**,** Princeton, New Jersey, United States of America; Ferrarotto Hospital, University of Catania, Italy

## Abstract

**Objective:**

To assess the extent to which the observed racial disparities in cardiac revascularization use can be explained by the variation across counties where patients live, and how the within-county racial disparities is associated with the local hospital capacity.

**Data Sources:**

Administrative data from Pennsylvania Health Care Cost Containment Council (PHC4) between 1995 and 2006.

**Study Design:**

The study sample included 207,570 Medicare patients admitted to hospital for acute myocardial infarction (AMI). We identified the use of coronary artery bypass graft (CABG) and percutaneous coronary intervention (PCI) procedures within three months after the patient’s initial admission for AMI. Multi-level hierarchical models were used to determine the extent to which racial disparities in procedure use were attributable to the variation in local hospital capacity.

**Principal Findings:**

Blacks were less likely than whites to receive CABG (9.1% vs. 5.8%; p<0.001) and PCI (15.7% vs. 14.2%; p<0.001). The state-level racial disparity in use rate decreases for CABG, and increases for PCI, with the county adjustment. Higher number of revascularization hospitals per 1,000 AMI patients was associated with smaller within-county racial differences in CABG and PCI rates. Meanwhile, very low capacity of catheterization suites and AMI hospitals contributed to significantly wider racial gap in PCI rate.

**Conclusions:**

County variation in cardiac revascularization use rates helps explain the observed racial disparities. While smaller hospital capacity is associated with lower procedure rates for both racial groups, the impact is found to be larger on blacks. Therefore, consequences of fewer medical resources may be particularly pronounced for blacks, compared with whites.

## Introduction

The considerable geographic variation in medical spending has raised widespread concerns about the inefficiency of the United States healthcare system. Although the abundance of medical resources, such as specialty physicians and hospital beds, have been shown to be associated with greater treatment intensity for chronically ill patients [1]–[3], studies disagree on whether higher utilization translates into better patient outcomes [4], [5]. This phenomenon has led to various policy initiatives that have focused on provider incentives to curtail the overuse of resource-intensive services, such as reducing Medicare reimbursement for hospitals and surgeons [6], and strengthening regulatory approaches to limit new hospital services. On the other hand, many are concerned that aggressive policy changes could lead to a shortage of surgeons and hospitals, consequently threatening patient access to care [7], [8]. To the extent that minority populations are particularly vulnerable to inadequate access to care, it is important to understand the unintended effects of the changes in medical resources on racial disparities of medical utilization.

Cardiac revascularization procedures, comprising coronary artery bypass graft (CABG) and percutaneous coronary interventions (PCI) are the most commonly performed medical procedures in the U.S. About 1 million adults undergo CABG and PCI annually [9], with more than half of these procedures performed for elderly patients over age 65 [10]. Large racial inequalities in the provision of cardiac revascularization procedures are consistently documented in the literature, and such differences cannot be fully explained by heterogeneity in patient clinical presentations, socioeconomic status, insurance coverage, and preferences [11]–[19]. Previous studies have pointed out that racial disparities are often attributed to where patients live: the medical utilization among minority populations are lower on average because they tend to cluster in geographic areas with lower utilization rates for all racial groups [20], [21]. While these studies underscored the importance of geography in the measurement of racial disparity, there has been limited discussion of the potential causes of within-area racial disparities. Some researchers found evidence that local factors such as lower income and higher degree of residential segregation could lead to wider racial gaps in medical utilization [21], yet little is known about the influence of hospital capacity.

In this study, we examined the relationship between local medical resources and racial disparity in cardiac revascularization utilization by analyzing the use of CABG and PCI among Pennsylvania Medicare beneficiaries from 1995 to 2006. Specifically, we sought to address two questions: (1) To what extent can the state-level racial disparities in the rates of CABG and PCI be explained by variations in procedure rates across counties? (2) Are the within-county racial disparities in procedure rates attributable to local hospital capacity?

## Methods

### Data and Sample

This study performed a retrospective analysis using the administrative data from Pennsylvania Health Care Cost Containment Council (PHC4). The PHC4 collects detailed patient demographic and utilization information, including age, gender, race, diagnosis and procedure codes, diagnosis related groups (DRGs), and source of admission, for all hospital discharges occurring in all Pennsylvania hospitals.

The sample for this analysis was comprised of Medicare beneficiaries (patients with Medicare listed as the primary expected payer) admitted with a new primary diagnosis of AMI, defined as the International Classification of Diseases, Ninth Revision, Clinical Modification (ICD-9-CM) code of 410.xx, between 1995 and 2006. We focused on the Medicare population in order to eliminate the substantial heterogeneity in health insurance coverage that affects the likelihood of receiving surgeries. Patients who were admitted with the same illness in the prior year or for subsequent episodes of care (ICD-9-CM 410.x2) were excluded. This analysis was limited to AMI patients who resided in Pennsylvania, and who were Black or White. Other racial groups including Asian or Pacific Islander, Hispanic American Indian or Eskimo, and other or unknown race were excluded due to low numbers of observations. Collectively, these patients accounted for 7.7% of all Pennsylvanian AMI patients. Patients were linked over time via a unique patient ID, which enabled us to identify the procedure used following the first AMI admission.

### Ethics Statement

The PHC4 data are hospital discharge data collected primarily for administrative and billing purposes. Written consent was given by the patients for their information to be stored in the hospital database and used for research. All records are stripped of personal identifiers. According to the HHS regulations (Code of Federal Regulations, title 45, sec. 46.1), this research is exempt from the HHS policy, and thus IRB approval is not required.

### Outcome Measures and Covariates

The outcome measures were the individual-level use of CABG and PCI procedures within three months of the AMI diagnosis. The three-month cutoff is often use by prior studies to examine the process of care for AMI patients [22], [23]. Procedure use was identified by the appearance of ICD-9-CM procedure codes 36.1X for CABG, and 36.01, 36.02, 36.05, 36.07, 36.09, or 00.66 for PCI.

The primary independent variable was patient race group, coded as one if the patient is black. Other patient sociodemographic characteristics included gender, age group (<65, 65–75 and >75), primary payer type (Medicare managed care plan, or others), secondary expected payer type (Medicaid, private insurance, or others), and log of median household income at the patient’s zip code of residence, which was obtained by linking patient zip code to the US Census 2000 Summary File 3. To adjust for patient illness severity and preexisting conditions, we included the count of comorbid conditions according to AHRQ Elixhauser comorbidity diagnostic categories [24] and dummy variables of major clinical indications affecting the quality of CABG/PCI procedures (hypertension, heart failure, cardiogenic shock, cancer, renal failure, other coronary artery diseases, history of CABG/PCI procedure). We also considered the source of admission: whether the patient was admitted from an emergency department, and whether the patient was transferred from another health care facility (hospital, skilled nursing facility, intermediate care facility, or assisted living facility); the reference group consisted of patients directly admitted to hospital.

In the main analysis, we used the number of revascularization hospitals per 1,000 AMI patients in each county to measure local hospital capacity. Following prior literature, we defined revascularization hospitals as those that performed at least 5 CABG or PCI procedures annually [12]. Hospitals that performed PCI but did not perform CABG were also counted because such facilities represented a nontrivial proportion of access to cardiac care services in nonurban areas [25]. To detect any non-linear relationship between the hospital capacity and procedure rates, we coded the number of hospitals per capita into quintile categories.

### Statistical Analyses

We first compared the average procedure rates and baseline characteristics between the two racial groups using tests adjusted for clustering of patients within counties. We then performed a series of regression analyses on the use of CABG and PCI procedures. The initial model is a linear probability regression of the incidence of procedures on the race indicator and year dummies, which control for secular trends of procedure rates (the coefficient for the race dummy variable therefore identified the “raw” differences in procedure rates between blacks and whites). Second, we adopted a richer specification that adjusted for heterogeneity in the above-mentioned patient sociodemographics and clinical characteristics. Third, to further investigate how the estimated racial differences in procedure use might be affected by county-level factors, we estimated a multi-level hierarchical model with county as random effects and patient-level characteristics as fixed effects. We employed a hierarchical model because it takes into account the fact that revascularization procedure use for patients within the same county may be correlated, and therefore allows us to examine differences in procedure use among patients within county, conditional on patient-level characteristics and state-wide time trend. Likelihood ratio tests were used to compare the fit of the hierarchical model over ordinary least square model. Finally, we estimated a cross-level hierarchical model that additionally included the random effects of county-level hospital capacity, as well as the interaction between hospital capacity and the patient race group. This model assumes that not only the mean procedure rates, but also the within-county racial differences, vary by county’s hospital capacity. It allows us to separately identify the average within-county racial difference, the average impact of hospital capacity on the use rate, and most importantly, the extent to which the racial difference widens or narrows with the increase of local hospital capacity.

We initially estimated hierarchical models with binomial logit link; however, such models were computationally infeasible due to the large sample size. Based on previous studies, which demonstrated that the estimated marginal effects from linear probability model and logit model tend to converge with large sample size [26], [27], we used linear hierarchical models as an alternative. To ascertain that the linear models yield similar results as logit models, we also estimated logit models with county fixed effects, which allowed for a separate intercept for each of the 67 counties in our sample. In all models the standard errors were adjusted for clustering of patients within counties.

We performed sensitivity analyses to examine the robustness of the findings. First, we considered the number of catheterization suites in each county as an alternative measure of hospital capacity. The availability of cardiac catheterization procedures in a hospital was identified from the AHA data. We repeated the cross-level model estimation, replacing quintiles of revascularization hospitals per capita with quintiles of catheterization suites per capita. Second, we examined whether racial differences in procedure use were also attributable to non-procedural hospital resource for the AMI population, which was defined as the number of AMI hospitals (treating at least five AMI cases during a year) per capita in each county. Lastly, we explored the potential impact of physician supply. Using unique IDs of operating surgeons from the data set, we calculated the number of CABG surgeons and PCI interventional cardiologists per capita in each county, and then repeated the cross-level estimation.

## Results

Our study sample included 195,043 (94.7%) white and 10,887 (5.3%) black Medicare enrollees, who were initially hospitalized for AMI in 67 counties and 234 hospitals between 1995 and 2006. Within three months of the admission, 9% of these patients underwent CABG procedure and 15.7% underwent PCI procedure (Table 1). The use rates of both CABG and PCI were lower among black patients. As compared with the proportion of white patients, a higher proportion of black patients were female, aged below 75, enrolled in Medicare managed care plans, and living in areas with lower median household income. The black population represented a larger proportion of patients who were dual-eligible for the Medicaid program, a finding that probably reflects a higher percentage of low-income Medicare enrollees among blacks. In regard to the admission source, black patients were more likely to be admitted through the emergency department, while white patients were more likely to be transferred from other facilities. Despite the lower procedure rates, black patients were significantly sicker than white patients upon admission: they were more likely to have two or more comorbidities, and have higher rates of preexisting hypertension, congestive heart failure, diabetes, renal failure, and cancer.

**Table 1:** Descriptive Statistics by Race^a^.

	Total	Whites	Blacks	
	(n = 207,570)	(n = 195,043)	(n = 10,887)	*P* Value^b^
Dependent Variable				
CABG within 3 months	9.0	9.1	5.8	<0.0001
PCI within 3 months	15.6	15.7	14.2	<0.0001
Patient demographics and clinical history				
Male	48.3	48.6	42.6	<0.0001
Age 64-	8.1	7.6	16.9	<0.0001
Age 65–74	34.1	33.9	38.3	<0.0001
Age 75+	57.8	58.5	44.8	<0.0001
Primary payer: Medicare managed care	16.2	15.8	23.2	<0.0001
Second payer: Medicaid	5.5	4.8	18.1	<0.0001
Second payer: private insurance	41.9	42.9	25.1	<0.0001
Mean log of household income^c^ (SE)	10.6 (0.30)	10.6 (0.29)	10.3 (0.36)	<0.0001
Transferred admission	14.1	14.4	7.7	<0.0001
Emergency admission	72.1	71.7	80.5	<0.0001
Hypertension	43.8	43.1	55.4	<0.0001
Congestive heart failure	0.6	0.6	1.0	<0.0001
diabetes	26.6	26.2	34.4	<0.0001
Renal failure	5.8	5.4	11.9	<0.0001
cancer	2.6	2.6	3.4	<0.0001
Cardiogenic shock	4.7	4.8	3.7	<0.0001
Other coronary artery diseases	61.0	61.2	55.9	<0.0001
CABG	3.2	3.2	2.4	<0.0001
PCI	4.5	4.5	3.8	<0.0001
Elixhauser 0	19.4	19.8	12.3	<0.0001
Elixhauser 1	33.6	33.8	29.5	<0.0001
Elihauser 2	28.1	28.0	30.9	<0.0001
Elihauser 3+	18.8	18.4	27.3	<0.0001
Number of revascularization hospitals/1000 AMI patients				
First quintile: 0–0.84	25.9	27.1	4.1	<0.0001
Second quintile: 0.85–2.03	14.2	14.6	6.3	<0.0001
Third quintile: 2.04–2.96	20.0	20.0	20.6	0.16
Fourth quintile: 2.97–3.89	20.1	19.1	38.7	<0.0001
Fifth quintile: 3.90+	19.8	19.2	30.3	<0.0001

Abbreviations: CABG, coronary artery bypass graft; PCI, percutaneous coronary intervention; SE, standard error.

a Data are presented as percentages unless otherwise indicated.

b *t* Tests.

c Household income is abstracted at level of zip code.

Table 2 shows the use of CABG and PCI among blacks and whites in ten counties with the highest black population densities in the study sample. In seven of the ten counties, the rate of CABG within three months of the initial AMI diagnosis was significantly lower for blacks than for whites. In contrast, the rate of PCI within three months was not significantly different among blacks and whites in six of the ten counties. In the remaining four counties, Philadelphia had a significantly higher PCI rate among blacks than among whites.

**Table 2:** Use of CABG and PCI in Selected Counties, By Race.

	CABG Within Three Months (%)			PCI Within Three Months (%)		
County	White	Black	Difference	White	Black	Difference
Philadelphia	7.0	5.6	1.4^c^	12.3	13.4	−1.2^b^
Dauphin	10.0	8.4	1.6	20.0	19.9	0.1
Delaware	8.5	5.2	3.3^c^	13.3	11.2	2.1^a^
Allegheny	9.3	6.2	3.1^c^	17.1	15.1	2.1^b^
Chester	7.9	4.3	3.6^b^	15.9	16.2	−0.3
Beaver	9.5	5.3	4.3^b^	22.0	16.3	5.7^b^
Monroe	7.8	4.2	3.5^c^	15.1	13.6	1.5
Erie	12.0	2.5	9.4^c^	17.2	16.9	0.3
Mercer	7.1	6.4	0.7	15.7	15.4	0.3
Washington	11.1	10.2	0.9	20.9	18.5	2.4

a p<0.1;

b p<0.05;

c p<0.01 (two-tailed tests).

In Tables 3 and 4, we examine the first research question which looks at the extent to which state-level racial disparities in CABG and PCI rates can be explained by variations in procedure rates across counties. Table 3 reports the adjusted racial disparities in the use of CABG. Coefficients represent percentage differences in procedure rates. Adjusted for year trend, the likelihood of undergoing CABG was, on average, 3.2 percentage points (95% confidence interval (CI): −0.038–−0.027), lower for blacks than for whites. Given that the time-adjusted average use rate of CABG was 9.1% among whites, this estimate means that blacks were 35.2% less likely to undergo CABG. Adjusting for patient characteristics significantly reduced the racial difference to 2.4 percentage points (95% CI: −0.034–−0.014), which amounted to 26.4% based on the risk-adjusted rate for whites. Controlling for county random effects further reduced the magnitude of disparity to 1.9 percentage points (95% CI: −0.027–−0.011), suggesting that blacks and whites in the same county were treated more similarly than we would have assumed based on state-level risk-adjusted difference. The improvement in fit compared to the linear model was significant (χ^2^ = 613.54, p<.0001). The FE logit model estimated virtually the same results as the random effects model. This set of results indicates that a significant proportion of observed racial disparity in the CABG rate was due to black patients, on average, living in counties with lower CABG rates among both blacks and whites.

**Table 3:** Factors Associated With CABG Use Within Three Months Among Newly Diagnose AMI patients.

	Year Adjusted*		+ Patient Characteristics		+ County Random Effects		FE Logit Model	
	Coefficient (95% CI)		Coefficient (95% CI)		Coefficient (95% CI)		Coefficient (95% CI)	
Black	−0.032^c^	(−0.038–−0.027)	−0.024^c^	(−0.034–−0.014)	−0.019^c^	(−0.027–−0.011)	−0.022^c^	(−0.032–−0.012)
Male			0.029^c^	(0.025–0.032)	0.029^c^	(0.027–0.032)	0.028^c^	(0.026–0.031)
Age 65–74			0.018^c^	(0.011–0.024)	0.018^c^	(0.013–0.022)	0.016^c^	(0.01–0.022)
Age 75+			−0.043^c^	(−0.050–−0.036)	−0.042^c^	(−0.047–−0.037)	−0.041^c^	(−0.047–−0.036)
Primary payer: Medicare managed care			0.002	(−0.004–0.008)	0.004^b^	(0.000–0.008)	0.004^a^	(0–0.008)
Second payer: Medicaid			−0.024^c^	(−0.033–−0.014)	−0.022^c^	(−0.028–−0.017)	−0.024^c^	(−0.032–−0.015)
Second payer: private insurance			−0.002	(−0.011–0.008)	−0.002	(−0.004–0.001)	−0.001	(−0.009–0.007)
Log household income (zip code level)			0.006	(−0.005–0.018)	0.005^a^	(−0.000–0.011)	0.003	(−0.003–0.009)
Transferred admission			0.060^c^	(0.051–0.070)	0.061^c^	(0.057–0.066)	0.039^c^	(0.032–0.047)
Emergency admission			−0.036^c^	(−0.045–−0.028)	−0.040^c^	(−0.044–−0.037)	−0.043^c^	(−0.051–−0.035)
Hypertension			0.019^c^	(0.014–0.024)	0.019^c^	(0.016–0.022)	0.018^c^	(0.013–0.023)
Congestive heart failure			−0.019	(−0.042–0.004)	−0.021^b^	(−0.037–−0.005)	−0.021	(−0.05–0.008)
Diabetes			−0.000	(−0.005–0.004)	−0.000	(−0.003–0.003)	0	(−0.004–0.005)
Renal failure			−0.015^c^	(−0.021–−0.008)	−0.016^c^	(−0.021–−0.010)	−0.017^c^	(−0.023–−0.01)
Cancer			−0.038^c^	(−0.046–−0.029)	−0.037^c^	(−0.045–−0.029)	−0.041^c^	(−0.048–−0.033)
Cardiogenic shock			0.012^b^	(0.002–0.022)	0.010^c^	(0.005–0.016)	0.014c	(0.003–0.024)
Other coronary artery diseases			0.056^c^	(0.050–0.063)	0.055^c^	(0.052–0.057)	0.058^c^	(0.053–0.062)
Prior CABG			−0.083^c^	(−0.090–−0.077)	−0.084^c^	(−0.091–−0.078)	−0.079^c^	(−0.082–−0.077)
Prior PTCA			−0.043^c^	(−0.049–−0.038)	−0.044^c^	(−0.050–−0.038)	−0.039	(−0.044–−0.034)
Elixhauser 1			−0.000	(−0.004–0.004)	−0.001	(−0.004–0.003)	−0.002	(−0.006–0.002)
Elixhauser 2			0.000	(−0.006–0.007)	−0.001	(−0.005–0.004)	−0.001	(−0.007–0.006)
Elixhauser 3+			−0.007	(−0.015–0.002)	−0.008^c^	(−0.013–−0.003)	−0.007	(−0.016–0.001)
White, mean CABG use rate	0.091	(0.086–0.097)	0.091	(0.085–0.096)	0.084	(0.079–0.089)	0.090	(0.09–0.091)

* Standard errors in all models are adjusted for correlation in patients living in the same county.

a p<0.1;

b p<0.05;

c p<0.01 (two-tailed tests).

**Table 4:** Factors Associated With PCI Use Within Three Months Among Newly Diagnose AMI patients.

	Year Adjusted*		+ Patient Characteristics		+ County Random Effects		FE Logit Model	
	Coefficient (95% CI)		Coefficient (95% CI)		Coefficient (95% CI)		Coefficient (95% CI)	
Black	−0.019^c^	(−0.028–−0.010)	0.002	(−0.010–0.014)	−0.011^a^	(−0.023–0.001)	0	(−0.017–0.016)
Male			0.003^a^	(−0.000–0.007)	0.004^b^	(0.001–0.007)	0.002	(−0.001–0.005)
Age 65–74			−0.027^c^	(−0.034–−0.020)	−0.029^c^	(−0.034–−0.023)	−0.029	(−0.035–−0.023)
Age 75+			−0.115^c^	(−0.124–−0.106)	−0.116^c^	(−0.122–−0.111)	−0.115^c^	(−0.122–−0.108)
Primary payer: Medicare managed care			0.007	(−0.011–0.025)	0.009^c^	(0.005–0.014)	0.008^c^	(−0.003–0.018)
Second payer: Medicaid			−0.030^c^	(−0.044–−0.016)	−0.024^c^	(−0.030–−0.017)	−0.023^c^	(−0.035–−0.011)
Second payer: private insurance			0.004	(−0.012–0.020)	0.011^c^	(0.007–0.014)	0.011^c^	(0–0.022)
Log household income (zip code level)			0.032^c^	(0.011–0.053)	0.013^c^	(0.006–0.020)	0.011^c^	(0.003–0.018)
Transferred admission			0.069^c^	(0.052–0.085)	0.073^c^	(0.067–0.079)	0.057^c^	(0.044–0.07)
Emergency admission			−0.046^c^	(−0.063–−0.029)	−0.051^c^	(−0.055–−0.046)	−0.052^c^	(−0.063–−0.04)
Hypertension			0.056^c^	(0.051–0.061)	0.055^c^	(0.052–0.059)	0.057^c^	(0.053–0.061)
Congestive heart failure			−0.063^c^	(−0.076–−0.051)	−0.067^c^	(−0.086–−0.047)	−0.107	(−0.125–−0.09)
Diabetes			−0.001	(−0.005–0.003)	0.000	(−0.004–0.004)	0.002	(−0.002–0.007)
Renal failure			−0.058^c^	(−0.064–−0.051)	−0.061^c^	(−0.067–−0.054)	−0.054^c^	(−0.061–−0.048)
Cancer			−0.018^c^	(−0.026–−0.011)	−0.017^c^	(−0.026–−0.007)	−0.023^c^	(−0.032–−0.014)
Cardiogenic shock			−0.040^c^	(−0.053–−0.028)	−0.044^c^	(−0.051–−0.037)	−0.043^c^	(−0.055–−0.03)
Other coronary artery diseases			0.103^c^	(0.093–0.113)	0.097^c^	(0.094–0.101)	0.104^c^	(0.097–0.111)
Prior CABG			−0.029^c^	(−0.037–−0.020)	−0.030^c^	(−0.038–−0.021)	−0.022^c^	(−0.029–−0.016)
Prior PTCA			0.065^c^	(0.052–0.077)	0.059^c^	(0.052–0.066)	0.047^c^	(0.039–0.056)
Elixhauser 1			−0.051^c^	(−0.057–−0.045)	−0.052^c^	(−0.057–−0.048)	−0.065^c^	(−0.07–−0.06)
Elixhauser 2			−0.085^c^	(−0.093–−0.077)	−0.087^c^	(−0.092–−0.082)	−0.098^c^	(−0.104–−0.092)
Elixhauser 3+			−0.115^c^	(−0.125–−0.105)	−0.117^c^	(−0.123–−0.110)	−0.124^c^	(−0.131–−0.117)
White, mean PTCA use rate	0.157	(0.145–0.168)	0.156	(0.146–0.166)	0.140	(0.131–0.150)	0.156	(0.155–0.157)

* Standard errors in all models are adjusted for correlation in patients living in the same county.

a p<0.1;

b p<0.05;

c p<0.01 (two-tailed tests).

Among other covariates, men on average had higher CABG rate than women; elderly Medicare patients aged between 65 and 74 were more likely to undergo CABG than those under 65; and those who were above 75 had a significantly lower rate. Having Medicaid listed as the secondary expected payer was associated with a lower CABG rate, possibly reflecting that low-income senior patients who were dually eligible for Medicare and Medicaid have limited access to care [28]. Patients transferred from other facilities were more likely to receive CABG, while patients admitted through the emergency department were less likely to. Conditional on patient sociodemographic presentation, most of the clinical indications were significantly associated with higher likelihood of receiving CABG.

Table 4 shows that while risk-adjustment erased the raw difference in PCI rates (−0.019 (−0.028–−0.01) vs. 0.002 (−0.01–0.014)), further adjusting for the county-level variation led to widened disparity (−0.011 (−0.023–0.001)), although the estimate was marginally significant. Again, the likelihood ratio test indicates a better model fit using county-specific random effects (χ^2^ = 1702.15, p<.0001). The FE logit results suggest that the risk-adjusted difference in PCI rate did not change with the addition of county fixed effects. These results combined suggest that, on average, blacks might be slightly less likely to undergo PCI than whites within the same county, but that the difference was offset by the fact that blacks cluster in counties with relatively higher PCI rates among both blacks and whites.

Table 5 explores the second research question, namely, whether the within-county racial disparities are attributable to local hospital capacity. Counties were stratified into five quintiles according to their hospital capacity, which was defined as the number of revascularization hospitals per 1,000 AMI patients. Consistent with our understanding, results indicate that the local hospital capacity contributed to higher procedure rates for both racial groups and reduced the gap between whites and blacks. Although the confidence interval for each of these categorical variables overlapped with each other, we focus on comparing the magnitudes of the coefficients in interpreting the results. Estimates from both the hierarchical model and FE logit model indicate that conditional on patient clinical and sociodemographic factors, those living in counties with the larger hospital capacity were more likely to undergo CABG and PCI. The coefficients on the interaction terms suggest that the effect of county-level hospital capacity on racial disparity was not linear; however, overall, increased hospital capacity was associated with a gradient reduction in the within-county racial difference in CABG and PCI rates. For instance, estimates from hierarchical models show that, all else being equal, black patients living in counties of the lowest hospital capacity (the first quintile) were 3.9 percentage points (95% CI: −0.065–−0.013) less likely to undergo CABG and 4.2 percentage points (95% CI: −0.074–−0.010) less likely to undergo PCI than their white counterparts, while blacks living in counties of the largest hospital capacity (the fifth quintile) were 2.3 percentage points (95% CI: −0.034–−0.011) less likely to undergo CABG and had equal PCI rates with white patients (point estimate: 0.009, 95% CI: −0.003–0.022).

**Table 5:** Differences in CABG and PCI Use in Association With County-level Revascularization Hospital Capacity.

	CABG Within Three Months				PCI Within Three Months			
	Hierarchical Model		FE Logit model		Hierarchical Model		FE Logit model	
	Coefficient (95% CI)		Coefficient (95% CI)		Coefficient (95% CI)		Coefficient (95% CI)	
Revascularization Hospitals Per Capita								
First quintile	–	–	–	–	–	–	–	–
Second quintile	0.011^c^	(0.003–0.019)	0.004	(−0.006–0.014)	0.026^c^	(0.016–0.036)	0.014	(−0.007–0.034)
Third quintile	0.013^c^	(0.006–0.019)	0.01^a^	(0–0.019)	0.029^c^	(0.021–0.038)	0.018	(−0.004–0.04)
Fourth quintile	0.015^c^	(0.009–0.022)	0.012^a^	(0.002–0.022)	0.037^c^	(0.028–0.045)	0.027	(0.004–0.049)
Fifth quintile	0.019 c	(0.013–0.026)	0.017^c^	(0.008–0.027)	0.038^c^	(0.030–0.047)	0.028	(0.006–0.05)
Interaction Effect								
Black, first quintile	−0.039^c^	(−0.065–−0.013)	−0.042^c^	(−0.064–−0.02)	−0.042^c^	(−0.074–−0.010)	−0.059^c^	(−0.077–−0.042)
Black, second quintile	−0.026^b^	(−0.048–−0.004)	−0.028^c^	(−0.04–−0.016)	−0.018	(−0.044–0.009)	−0.022^b^	(−0.043–−0.002)
Black, third quintile	−0.031^c^	(−0.044–−0.018)	−0.028^c^	(−0.032–−0.024)	−0.013^a^	(−0.028–0.002)	−0.013^b^	(−0.023–−0.002)
Black, fourth quintile	−0.024^c^	(−0.035–−0.014)	−0.017^c^	(−0.029–−0.005)	0.005	(−0.007–0.016)	0.009	(−0.007–0.024)
Black, fifth quintile	−0.023^c^	(−0.034–−0.011)	−0.016^c^	(−0.026–−0.006)	0.009	(−0.003–0.022)	0.014^c^	(0.006–0.022)

a p<0.1;

b p<0.05;

c p<0.01 (two-tailed tests).

Because the availability of diagnostic catheterization, a standard procedure used to determine a patient’s need for revascularization treatment, may also affect revascularization use, we examined the impact of the number of catheterization suites per capita as an alternative measure of hospital capacity. Table 6 shows that higher quintile of catheterization capacity was not significantly associated with greater likelihood of receiving CABG, nor was it associated with narrowed racial difference in CABG rates (i.e., the magnitudes of cross-level interaction effects did not show a decreasing trend across quintile groups). For PCI, the within-county racial gap was sizable and significant only in counties with the lowest catheterization capacity.

**Table 6:** Differences in CABG and PCI Use in Association With County-level Catheterization Hospital Capacity.

	CABG Within Three Months				PCI Within Three Months			
	Hierarchical Model		FE Logit model		Hierarchical Model		FE Logit model	
	Coefficient (95% CI)		Coefficient (95% CI)		Coefficient (95% CI)		Coefficient (95% CI)	
Catheterization Suites Per Capita								
First quintile	–	–	–	–	–	–	–	–
Second quintile	−0.004	(−0.010–0.001)	−0.007	(−0.015–0.002)	−0.004	(−0.010–0.003)	−0.004	(−0.013–0.006)
Third quintile	−0.003	(−0.008–0.003)	−0.005	(−0.014–0.004)	0.005	(−0.002–0.013)	0.006	(−0.008–0.02)
Fourth quintile	−0.006^b^	(−0.012–−0.001)	−0.008	(−0.023–0.007)	0.008^b^	(0.001–0.015)	0.01	(−0.006–0.027)
Fifth quintile	−0.003	(−0.009–0.003)	−0.005	(−0.017–0.008)	0.013^c^	(0.006–0.020)	0.016^a^	(0–0.033)
Interaction Effect								
Black, first quintile	−0.030^a^	(−0.063–0.003)	−0.036^c^	(−0.056–−0.015)	−0.048^b^	(−0.089–−0.008)	−0.045^c^	(−0.069–−0.021)
Black, second quintile	−0.022^c^	(−0.035–−0.009)	−0.025^c^	(−0.036–−0.014)	0.002	(−0.014–0.018)	0.012	(−0.003–0.027)
Black, third quintile	−0.007	(−0.020–0.006)	−0.011	(−0.026–0.004)	−0.007	(−0.024–0.009)	0.003	(−0.008–0.014)
Black, fourth quintile	−0.02^c^	(−0.034–−0.009)	−0.024^c^	(−0.04–−0.008)	−0.004	(−0.020–0.012)	−0.003	(−0.024–0.019)
Black, fifth quintile	−0.016^b^	(−0.031–−0.001)	−0.024^c^	(−0.038–−0.01)	−0.012	(−0.030–0.006)	−0.007	(−0.024–0.009)

a p<0.1;

b p<0.05;

c p<0.01 (two-tailed tests).

Similarly, Table 7 shows that the racial difference in CABG rates remained similar in counties with different levels of AMI hospital capacity, and that the difference in PCI rate was larger and more significant in counties with the lowest AMI hospital capacity. Thus, we must conclude that very low capacity of catheterization suites and AMI hospitals contributed to wider racial gap in the use of PCI.

**Table 7:** Differences in CABG and PCI Use in Association With County-level AMI Hospital Capacity.

	CABG Within Three Months				PCI Within Three Months			
	Hierarchical Model		FE Logit model		Hierarchical Model		FE Logit model	
	Coefficient (95% CI)		Coefficient (95% CI)		Coefficient (95% CI)		Coefficient (95% CI)	
AMI Hospitals Per Capita								
First quintile	–	–	–	–	–	–	–	–
Second quintile	0.001	(−0.003–0.006)	0.001	(−0.005–0.008)	0.011^c^	(0.005–0.017)	0.011^b^	(0.002–0.02)
Third quintile	0.010^c^	(0.005–0.015)	0.011^b^	(0.001–0.02)	0.011^c^	(0.005–0.018)	0.011^b^	(0–0.021)
Fourth quintile	0.004	(−0.002–0.010)	0.007^c^	(−0.001–0.015)	0.017^c^	(0.010–0.024)	0.016^a^	(−0.001–0.033)
Fifth quintile	0.004	(−0.003–0.011)	0.009^b^	(0–0.018)	0.012^b^	(0.003–0.020)	0.012	(−0.004–0.029)
Interaction Effect								
Black, first quintile	−0.018^b^	(−0.037–0.000)	−0.03^c^	(−0.043–−0.018)	−0.027^b^	(−0.050–−0.003)	−0.018^a^	(−0.039–0.003)
Black, second quintile	−0.023^c^	(−0.037–−0.010)	−0.025^c^	(−0.04–−0.011)	−0.01	(−0.029–0.008)	0.001	(−0.022–0.024)
Black, third quintile	−0.020^c^	(−0.032–−0.008)	−0.019^c^	(−0.032–−0.006)	−0.013	(−0.030–0.003)	0	(−0.023–0.023)
Black, fourth quintile	−0.019^b^	(−0.034–−0.004)	−0.016^b^	(−0.029–−0.002)	0.001	(−0.019–0.021)	0.009	(−0.006–0.024)
Black, fifth quintile	−0.024^c^	(−0.039–−0.010)	−0.022^c^	(−0.034–−0.011)	−0.006	(−0.025–0.013)	0.004	(−0.011–0.019)

a p<0.1;

b p<0.05;

c p<0.01 (two-tailed tests).

Finally, Table 8 investigates whether racial disparities in revascularization procedure use were also attributable to the level of physician supply. The sample was stratified by the number of CABG surgeons per capita in the CABG regressions and by the number of PCI interventionists per capita in the PCI regressions. Estimates in Table 8 indicate that while higher physician capacity was strongly correlated with higher CABG and PCI use in general, it was not systematically correlated with the size of racial gaps.

**Table 8:** Differences in CABG and PCI Use in Association With County-level Physician Supply.

	CABG Within Three Months				PCI Within Three Months			
	Hierarchical Model		FE Logit model		Hierarchical Model		FE Logit model	
	Coefficient (95% CI)		Coefficient (95% CI)		Coefficient (95% CI)		Coefficient (95% CI)	
Surgeons/Interventional Cardiologists Per Capita								
First quintile	–	–	–	–	–	–	–	–
Second quintile	0.014^c^	(0.007–0.020)	0.015^c^	(0.005–0.026)	−0.001	(−0.007–0.004)	0.004	(−0.004–0.012)
Third quintile	0.017^c^	(0.010–0.024)	0.021^c^	(0.008–0.035)	0.005	(−0.001–0.011)	0.008^a^	(−0.001–0.017)
Fourth quintile	0.009^b^	(0.000–0.017)	0.017^c^	(0.005–0.029)	0.008^b^	(0.000–0.016)	0.012^b^	(0.002–0.022)
Fifth quintile	0.019^c^	(0.009–0.028)	0.033^c^	(0.018–0.049)	0.018^c^	(0.008–0.027)	0.023^b^	(0.004–0.041)
Interaction Effect								
Black, first quintile	0.005	(−0.007–0.018)	−0.017^b^	(−0.034–0)	0.006	(−0.012–0.024)	0.01	(−0.002–0.023)
Black, second quintile	−0.015^b^	(−0.029–−0.001)	−0.024^c^	(−0.036–−0.013)	0.002	(−0.015–0.018)	0.005	(−0.015–0.025)
Black, third quintile	−0.019^a^	(−0.039–0.002)	−0.033^c^	(−0.047–−0.02)	−0.003	(−0.027–0.021)	−0.009^a^	(−0.019–0.001)
Black, fourth quintile	−0.008	(−0.031–0.014)	−0.023^b^	(−0.042–−0.003)	−0.020	(−0.045–0.006)	−0.013	(−0.036–0.009)
Black, fifth quintile	−0.012	(−0.039–0.015)	−0.016^a^	(−0.032–0.001)	−0.013	(−0.045–0.020)	−0.011	(−0.029–0.007)

a p<0.1;

b p<0.05;

c p<0.01 (two-tailed tests).

## Discussion

Persistent racial disparities are well recognized by policy makers and clinicians as a serious health system problem in need of correction. Using inpatient claims data of Pennsylvania Medicare beneficiaries from 1995 to 2006, we show that, first, county-variation helps explain the differential cardiac revascularization use between black and white AMI patients. The findings that the state-level racial disparity in CABG procedure decreases, and in PCI procedure increases, with county adjustment indicate that blacks are more likely to live in counties with lower CABG rates and higher PCI rates for both black and white populations. Such results are in agreement with earlier studies on the use of coronary interventions and other procedures such as knee arthroplasty and hip replacement, which documented wide variability of procedure use among racial groups both within and between geographic regions [20], [21], [29]. Building on prior literature, our finding highlights the importance of controlling for small-area variations when evaluating racial disparities in medical utilization.

Second, we find evidence that wider racial gaps in CABG and PCI use rates within counties is attributable to county-level hospital capacity, measured by the number of cardiac revascularization hospitals per capita. In particular, a large difference in PCI use rate was observed in counties with very low capacity of catheterization suites and AMI hospitals. Such findings resonate with previous studies, which showed evidence that differences in local medical resource supply are inversely correlated with racial disparities in in-hospital mortality rate [30], suggesting that consequences of fewer medical resources may be particularly pronounced for blacks, compared with whites.

There are several potential explanations for the finding that larger hospital capacity may have contributed to smaller racial gaps in procedure use. First, recent studies present evidence that black patients are less likely to follow up with recommended, more expensive treatments than whites [31], [32]. This problem may be worse in areas with medical resource shortages, because patients in those areas are more likely to have insufficient awareness of where to access the best care, and to face logistic obstacles such as transportation costs and lack of time [33]. Second, the lack of local medical resources may have disproportionately limited black patients’ ability to obtain referrals and to access high-quality care. Whether a patient obtains referrals for procedures depends to some extent on whether the patient is being managed by a cardiac specialist. However, minority patients are less likely than white patients to have access to specialists [34]–[36]. Given that the referral for cardiac procedures is often influenced by the availability of cardiac procedure service [37], [38], the disparity in access to specialty care may exacerbate the observed racial gaps in the use rates in counties with lower cardiac hospital capacity. Third, challenges around care coordination may also play a role in racial differences in procedure rates. Past studies suggest that intensive follow-up with primary care physicians (PCPs) can reduce racial disparities in chronic care management [39], [40], however, physicians caring for minority patients face more difficulties in coordinating care, spending adequate time with patients, and obtaining specialty care for their patients [41]. Moreover, care coordination tends to be more challenging in areas with lower medical capacity as physicians’ ability to function is further undermined by constrained resources.

Our analysis also finds a positive association between the relative availability of cardiac revascularization services and higher procedure rates, especially among whites. This finding supports existing research that documents large, unwanted variation in the utilization of specialty care that is closely linked to the local health care resource [42]. When evaluating procedures based on explicit appropriateness classifications, numerous clinical studies find evidence of procedural overuse among whites and underuse among blacks [15], [43], [44]. Our finding provides further evidence that the potential overutilization by whites may be driven by the local medical service supply.

The results of our study provide important policy implications for addressing the health disparities among minority populations. For discretionary surgical procedures, policy should not simply focus on equalizing black rates with white rates. Instead, the objective depends critically on whether the racial gap stems from excessive utilization among whites or underutilization among blacks [14], [15], [43]. Where evidence suggests underutilization among minorities, care coordination and referral practices must be improved to ensure minority patients are receiving appropriate care. Equally important is ensuring white patients are not overutilizing services simply because of their availability. In the era of evidence-based medicine, such variation speaks to the need to reduce subjective decision making by continuing education of practicing professionals on the usefulness and appropriateness of different treatments and the importance of following evidence-based guidelines.

However, since cardiac procedures are well-reimbursed and the current fee-for-service (FFS) payment system rewards providers based on volume rather than appropriate and efficient use, it is difficult to effectively reduce the overuse of cardiac procedures merely with clinical decision-support guidelines. We believe that the emerging models of bundled or episode-based payment system could potentially address both the overuse and underuse problems and improve the efficiency of care delivery. Unlike the FFS payment, which covers the services by each provider separately, bundled payment covers a certain clinical episode or a defined time period [45], thereby ensuring that the financial risk and benefit are shared by hospitals, physicians, and patients, and encouraging providers to provide appropriate and efficient care for all patients. A recent demonstration project conducted by Geisinger’s ProvenCare shows that the bundled payment model significantly reduced hospital spending on CABG during a five-year period [46]. Because performance incentives are an important part of the model, measures related to clinical quality, patient experience, and cost efficiency need to be established. Therefore, providers will need significant infrastructure including electronic health records (EHRs) to be able to provide the quality information necessary for obtaining rewards of improved efficiency.

An important limitation of this analysis is our focus on Medicare beneficiaries. Although this approach greatly eliminates heterogeneity in health coverage among non-Medicare patients, we are unable to ascertain the effects of geography on racial disparities among other populations. Others have suggested that patients with private insurance or those insured through the Medicaid program may face different levels of disparities in care [47], [48]. The second limitation is that the inpatient claims data used for this analysis are collected primarily for billing purposes, and thus clinical details are limited. As a result, although we attempt to control for patient comorbidities, our specification lacks measures of detailed medical history and laboratory results that are used by physicians in making treatment decisions. Third, because the smallest time unit in the PHC4 data is quarter, we are not able to examine treatment patterns in a shorter time frame such as 30-day procedure incidence. Finally, our findings can be generalized only to populations that are similar in their characteristics to those in Pennsylvania. Future research is needed to replicate our findings in states with large black rural population such as Mississippi and Louisiana to determine the disparity in utilization by race and the extent to which county-level medical capacity differences account for such disparity.
